# The Spanish Core Collection of Common Beans (*Phaseolus vulgaris* L.): An Important Source of Variability for Breeding Chemical Composition

**DOI:** 10.3389/fpls.2018.01642

**Published:** 2018-11-13

**Authors:** Ana Rivera, Marçal Plans, Josep Sabaté, Francesc Casañas, Joan Casals, Aurora Rull, Joan Simó

**Affiliations:** ^1^Miquel Agustí Foundation, Barcelona, Spain; ^2^Nestle Purina, St. Louis, MI, United States; ^3^Department of Agri-Food Engineering and Biotechnology, BarcelonaTech, Universitat Politecnica de Catalunya, Barcelona, Spain

**Keywords:** *Phaseolus vulgaris*, common bean, diversity, genebanks, gene pool, nutrient composition, protein concentration, seed color

## Abstract

The Iberian Peninsula is considered as a secondary center of diversity for the common bean, and the Spanish National Plant Genetic Resources Centre’s germplasm bank holds more than 3,000 Spanish accessions of *Phaseolus vulgaris* L. from which a core collection of 202 landraces has been selected. In order to encourage the use of this abundant resource, this study aimed to characterize genetic diversity, by measuring chemical composition in these core collections (in both the seed coat and cotyledon) using previously developed near infrared spectroscopy models. Crucially, these landraces in question all originated under similar agroclimatic conditions, allowing these field trials to be conducted in a single location without significantly altering the agronomic behavior of individual accessions. Using previously reported data, we also explored the correlations between chemical composition and culinary/sensory traits, as well as possible associations between chemical composition and seed coat color or gene pool (Middle American or Andean). The general Mahalanobis distance was >3 in only 11 of 1,950 estimations, confirming the robustness of the regression models previously developed. Variability was greater in seed coat than in cotyledon compounds and ranges for all compounds were wide: ash 34–94 g/kg, Ca 5–31 g/kg, dietary fiber 554–911 g/kg, Mg 2–4.4 g/kg, uronic acid 95–155 g/kg, protein 192–304 g/kg, starch 339–446 g/kg, amylose 208–291 g/kg, amylopectin 333–482 g/kg, and apparent amylose 241–332 g/kg. Accessions with white seed coats tended to be richer in ash, dietary fiber, uronic acid, and Ca, and accessions of the Middle American gene pool had on average 65% more Ca than the Andean gene pool. Strong genetic correlations were not identified between chemical and culinary/sensory traits. This is particularly positive with regards to plant breeding, as it means that synchronic improvement of nutritional composition and sensory traits is possible. The genetic diversity of chemical composition described in the Spanish core collection of beans therefore represents a promising opportunity to develop cultivars with superior nutritional profiles.

## Introduction

Dry beans (*Phaseolus vulgaris* L.) are an extremely important aliment, not only representing the main source of dietary protein for humans in several world regions but also contributing greatly to diet with starch, fiber, vitamins, and minerals (Hayat et al., 2014). Annual global production is presently approximately 26.5 million tons, most of which is used for human consumption (FAOSTAT, 2018). Although in the most developed countries, the importance of dried bean consumption has diminished with the increase of meat consumption, producing animal protein is far more expensive, and is generally considered unsustainable (Pimentel and Pimentel, 2003; Gonzalez et al., 2011). Red meat in particular also has negative effects on health, for example being associated with the development of cardiovascular disease and cancer (Micha et al., 2010; Pan et al., 2013; Gonzales et al., 2014; Ekmekcioglu et al., 2018). International organizations and specialists therefore recommend increasing the consumption of beans and other legumes to fulfill our nutritional needs and decrease inputs of food production (Leterme, 2002; WHO, 2015; FAO, 2016; De Ron et al., 2017; McDermott and Wyatt, 2017). To promote this change in diet, apart from optimizing crop yields, we also need to identify palatable varieties of beans and legumes that are both tied to our gastronomic cultures and provide the maximum amount of nutrients (especially protein). Breeding programs for beans have focused mainly on maximizing yield (Kelly et al., 1998; Singh, 1999) and improving resistance to both biotic and abiotic stresses (Ishitani et al., 2004; Miklas et al., 2005; Singh and Schwartz, 2009; Araújo et al., 2015). More recently, however, nutritional and sensory traits have also been added to the ideotypes of these crops (Vaz Patto et al., 2015).

Natural genetic variation for chemical composition in the cultivated genepool and wild crop relatives of *Phaseolus vulgaris* is particularly important as it offers us the opportunity to develop cultivars with superior nutritional profiles. For example, reported percentages of protein content range from 18 to 31%, from 50 to 76% for carbohydrates, and from 0.05 to 0.31% for Calcium (Ca) (Sathe et al., 1984a; Islam et al., 2002; Pinheiro et al., 2010; Hayat et al., 2014). This genetic diversity can fortunately be found conserved in agrosystems located in the centers of diversity for the species (*in situ* conservation) and in genebanks (*ex situ* conservation). Of more than 7.4 million accessions stored in approximately 1,750 genebanks (FAO, 2010), 260,000 belong to *Phaseolus vulgaris* L. Although significant efforts have been devoted to characterizing such germplasm collections for simple traits (mostly related to botanical aspects) (Wang et al., 2017) and for the most important agronomic traits (yield and resistance to pests and diseases) (Tanksley and McCouch, 1997), less is known about other important characteristics such as those related to nutritional, culinary, or sensory attributes (Smýkal et al., 2015). This is mainly due to the fact that these traits are quantitative and multigenic, with low heritability and strong genotype by environment interactions (GxE), thus making it difficult to assign a genotypic value for each accession. Moreover, the phenotyping of these traits is expensive in terms of both direct monetary input and human labor. However, the lack of phenotyping data available, regarding the accessions conserved *ex situ*, hinders utilization of this genetic diversity both in breeding programs and directly by farmers (Ramanatha Rao and Hodgkin, 2002; Hodgkin et al., 2003). Novel “omics” platforms enabling the massive analysis of the genome, transcriptome, proteome, and metabolome (Tanksley and McCouch, 1997; Prada, 2009; Langridge and Fleury, 2011), and information science (Michael et al., 2018), represent promising tools to find desirable agronomic alleles in seed banks and unlock the stored genetic diversity. The development of core collections that are representative of the genetic spectrum in the whole collection (van Hintum et al., 2000) has been proposed as an alternative to enable cost-effective characterization of the plant genetic resources held in genebanks. Initially, complex traits are phenotyped in the core collection, and if desirable qualities are found, similar accessions can then be identified in the whole collection (Prada, 2009). An example of this strategy is the Spanish core collection of beans of the Spanish National Plant Genetic Resources Center (CRF), comprising 202 accessions representative of the variability present in the total set of accessions collected in Spain. This collection was compiled based on location data (province, township, and altitude) and seed phenotype (color, shape, and size) (De la Rosa et al., 2000) and is an important resource because the Iberian Peninsula is considered a secondary center of diversity for *Phaseolus vulgaris* L. (Santalla et al., 2002). To date, several traits have been measured in this collection: flower, pod, and seed traits; growth habit; type of phaseolin and molecular markers (Pérez-Vega et al., 2009); resistance to pests and diseases [including halo blight (*Pseudomonas syringae*), common bacterial blight (*Xanthomonas campestris*) (Asensio et al., 2010), anthracnose (*Colletotrichum lindemuthianum*) (Pérez-Vega et al., 2006), and white mold (*Sclerotinia sclerotiorum*) (Pascual et al., 2010)]; and sensory and culinary traits (Rivera et al., 2016). However, nothing was known about the variability in chemical composition and nutritional potential of this collection. The main factors generally limiting substantial chemical composition studies and comparison of materials conserved in genebanks are the difficulties involved in analyzing so many samples. Nowadays, however, instrumental methods such as near infrared spectroscopy (NIRS) or nuclear magnetic resonance spectroscopy (Hacisalihoglu et al., 2010; Parlak and Güzeler, 2016) make it possible to analyze large collections of samples. With this objective, our team has developed NIRS predictive models for chemical composition and sensory traits (Plans et al., 2012, 2013, 2014), which show a robust capacity for accurate prediction.

The present study extends our previous work about the culinary and sensory traits of the accessions in the CRF’s core collection of common beans (Rivera et al., 2016). Here, we examine the collection to (i) describe the variability of chemical composition in the seed coat and cotyledon, (ii) compare and correlate this variability and its magnitude with the variability in sensory and culinary traits described in the previous study, and (iii) analyze the possible associations between chemical composition and seed coat color and the Middle American or Andean gene pool.

## Materials and Methods

### Plant Material and Field Trials

**FIGURE 1:**
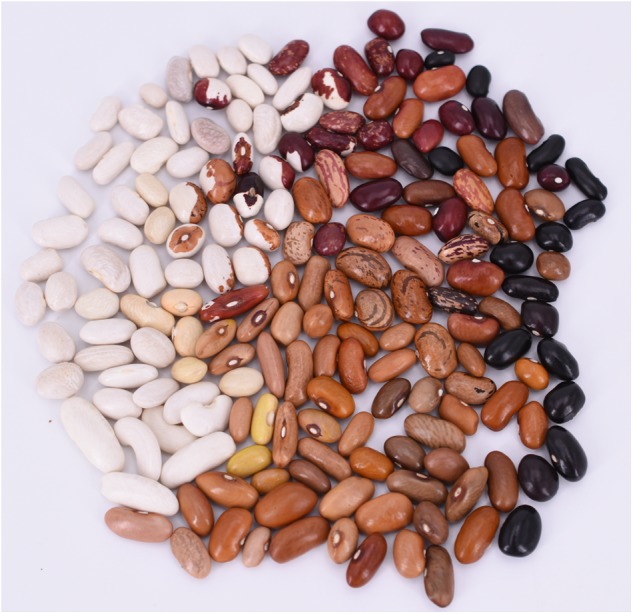
Photograph showing the variety of shapes and colors represented in the Spanish core collection of beans.

The 202 accessions from the Spanish core collection of common beans form a rich mosaic of colors and shapes (Figure 1), encompass 51 market classes (Santalla et al., 2001), and represent all areas of Spain where beans are cultivated. All of the seeds were sown in Sabadell (Northeast Spain: 41° 32′ 50.7″ N, 2° 4′ 14.7″ E), a location with loam soil with abundant Ca (6.35 mg/kg), low phosphorus (6 mg/kg), and a mild Mediterranean climate, which allows both short and long-cycle accessions to develop to maturity. We used a randomized two block design with 21 plants per block and accession (total 42 plants per accession). The experiment was conducted at a low density (29,167 plants/ha) to facilitate the individualized recording of data. A vertical plastic net was used to trellis the accessions with indeterminate growth. All plants were cultivated with the traditional management practices of the area, including drip irrigation and fert-irrigation application (NPK 110 kg/ha). Due to the lack of significance of the block factor for agronomic traits (data not shown), seeds from the two blocks were pooled in order to ensure sufficient sample was available for the analyses. Seeds from a total of 195 accessions were harvested and processed for analyses (seven accessions did not produce enough seeds for analyses).

### Sample Preparation

The seed coat accounts for less than 10% of the total dry matter of bean seeds and has a chemical composition distinctly different to that of the cotyledon (Singh et al., 1968; Moraghan et al., 2006). To ensure accurate information about the two fractions, seed coat, and cotyledons were analyzed separately. Seeds from each harvested accession (approximately 50 g) were soaked in 150 ml distilled water for 24 h, drained, dried with filter paper, and weighed. The seed coat was then manually separated from the cotyledon, and the two fractions were dried (60°C for 48 h). Finally, dried samples were ground to 0.4 mm in a laboratory mill (Perten 3100, Perten Instruments Inc., Springfeld, IL, United States). Ground samples were stored in polyethylene bags at 4°C until spectroscopic analysis was conducted.

### NIRS Recording to Estimate Chemical Composition

Near infrared spectroscopy is a well-established technique for determining the components of foods (Nicolaï et al., 2007). Models developed by means of multivariate analysis correlating physicochemical properties, and the spectra obtained with NIRS technology enable prediction of the value of a sample. In our study, we used NIRS predictive models previously developed by Plans et al. (2012), (2013), to estimate the following chemical components: ashes, Ca, magnesium (Mg), dietary fiber, and uronic acids in the milled seed coat; and protein, starch, amylose, amylopectin, and apparent amylose in the milled cotyledon. These traits were selected because of their relationship with sensory attributes driving consumer preferences, as reported in previous studies (Casañas et al., 2002, 2006; Mkanda et al., 2007). All results were expressed as g/kg dry matter. The estimations were calculated using regression models from NIRS measurements. Infrared spectra from the ground samples were recorded by a spectrophotometer (model 5000, Foss NIRSystems, Silver Spring, MD, United States) at every 2 nm between 1,100 and 2,500 nm, with a mean of 32 scans performed. Each sample was analyzed per triplicate, and the mean spectrum reading was used for calculations. The absorbance of each wavelength was transformed into Log (1/*R*), where *R* represents reflectance, due to this variable correlating better with chemical components than raw reflectance. To record spectra and import data, VISION software was used (version 2.51, Foss NIRSystems, Silver Spring, MD, United States).

### Reference Analysis

Twenty out of the accessions under study were randomly selected in order to perform chemical analysis. For each accession, three biological replicates were characterized for the chemical components. Ash, Ca, dietary fiber, Mg, and uronic acid were quantified by reference methods following the methodology described in Plans et al. (2012), while protein, starch, amylose, amylopectin, and apparent amylose were analyzed as described in Plans et al. (2013). These reference analyses were used to increase the domain of the chemical constituents of the NIRS model, thus improving its robustness as described by Barton et al. (2000).

### Calculating Chemical Composition

Two different approaches can be used to correlate NIRS data from samples with the constituents of interest (in this case, chemical composition): global models based on partial least squares regression (PLSR) and principal components regression (PCR) or local models based on the similarity of the unknown spectrum to the known spectra in a database. Classical regression techniques such as PLSR and PCR assume that the relationship between spectral and reference constituents is linear (Shenk and Westerhaus, 1991). The accuracy of NIRS in predicting chemical constituents is related to the structure and distribution of spectra in the calibration set, which must cover most of the possible variability in spectra encountered during routine analysis. For global PLSR models, increasing the domain of the calibration equation to include new diverse geographical and climatic areas, will require a larger number of representative samples in the calibration set. This, in turn, increases the complexity of the spectral reference model, making it necessary to compute more parameters to explain the variation in the constituents (Martens and Naes, 1989). The accuracy of prediction using global PLSR models usually decreases when the domain of the constituent increases.

Local calibration, also called memory-based regression, aims to overcome these difficulties. Since, in theory, an optimal prediction should be computed based on a specific calibration equation, we can use a small calibration dataset tailored to the unknown sample from a large database of spectra. This approach combines the advantage of global calibration (to cover a large constituent domain) and the accuracy obtainable with specific calibrations based on spectral similarities of a smaller set of samples. Various approaches can be used to calculate local models, with the most common algorithms being the classic similarities based on Mahalanobis distances (H) (Naes et al., 1990) and correlation coefficients (Shenk et al., 1997). Several authors found that local regression models significantly improved (20–30%) the standard error of prediction (SEP) compared to global calibration (Naes et al., 1990; Aastveit and Marum, 1993; Sinnaeve et al., 1994) in studies using sample selection and calibration methods based on PCR and PLSR.

We used global PLSR-derived correlations between NIR spectra and reference constituents (cotyledon: protein, starch, amylopectin, amylose, and apparent amylose; and seed coat: ash, calcium, dietary fiber, magnesium, and uronic acid). These models were previously developed using a wide range of genetic and environmental variability (with cultivation in many locations) (Plans et al., 2012, 2013). Furthermore, we increased the domain of the chemical constituents by using a set of 20 new accessions from the Spanish core collection of common beans. To avoid the problems inherent in using PLSR with increased domains, we used local regression (Ramirez-Lopez and Stevens, 2016) to correlate the spectra with the chemical referents. Local models were not developed using a single PLSR or PCR equation; rather a new calibration was created when an unknown sample was presented against the spectral database. The ability to predict unknown samples was based on global Mahalanobis distances (GH). This distance is normalized when measured in standard deviation units from the center of the selected set of samples. Based on a normal distribution, samples with a GH of greater than three would be classified as outliers and predictions in these samples would be considered questionable (Shenk et al., 1997).

### Statistical Analysis

To estimate the global variability for each trait, and the relationships between traits, we calculated their mean, and variance. Previous results on culinary, sensory, and external appearance traits obtained from the same samples (Rivera et al., 2016) were used to calculate the correlation with the results obtained in this study. Sensory traits were evaluated using regression models from NIRS measurements in ground cooked samples. These models were previously developed by using evaluations from a trained panel (Plans et al., 2014). By contrast, culinary traits were quantified directly by using a standardized cooking process, as described in Romero del Castillo et al. (2012). Correlation analysis was performed using the Pearson coefficient with the Bonferroni correction. For further analysis of the variability, the accessions were classified according to their seed coat color and origin gene pool (Middle American or Andean). The data concerning seed coat color were reported by Rivera et al. (2016). Seeds were visually evaluated and divided into two categories: white and colored (including: yellow, cream, gray brownish to greenish, brown, vinous brown, black, ochre, purple, rosy, bicolor, and tricolor). The origin gene pool was previously described by PérezVega et al. (2009), following study of the phaseolin protein pattern and 11 molecular markers [two sequence characterized amplified regions (SCAR) and nine simple sequence repeats (SSR)] in the collection. For each type of classification, analysis of variance (ANOVA) was carried out based on the linear model y*_ij_* = μ + α*_i_* + 𝜀*_ij_*, where y*_i_* was an individual level for a specific trait; μ was the grand mean; α_i_ was the effect of *i* group based on the type of seed coat color, or gene pool origin; and 𝜀*_ij_* was the random error for *i* groups with *j* replications of the model following a *N*∼(0, σ^2^). All factors were considered to be fixed. Finally, for seed coat color and gene pool classification, we performed normalized principal component analyses (PCA) and calculated 95% confidence ellipses around each cluster of accessions with the same category. We used R software (R Core Team, 2017); agicolae, PCAmethods, and ellipse packages for all statistical analyses (Murdoch and Chow, 2007; Stacklies et al., 2007; Mendiburu, 2017).

## Results

### Overall Variability in the Collection

The GH values obtained were extremely low and reflect the reliability of our estimations using the NIRs models. Only 11 from a total of 1950 estimations presented a GH value greater than 3. These values corresponded to amylopectin, and in each case were not considered in the subsequent computations. Furthermore, the mean GH values for individual traits all scored below GH < 1 (except amylopectin; GH < 1.3), thus highlighting the robustness of our data regarding the different chemical components in both the seed coat and cotyledon (Shenk et al., 1997).

The proportion of seed coat ranged from 4% to 10% (coefficient of variation, CV = 11.45%), and the proportion of cotyledon ranged from 90% to 96% (CV = 0.91%). For the chemical traits measured in the cotyledon, the CV ranged from 5.35% for starch to 10.97% for proteins. In general, CV from the seed coat components measured is higher than the CV from the cotyledon. In the seed coat, CV ranged from 8.31% for uronic acids to 39.07% for Ca (Table 1).

**Table 1:** Variability for the chemical compounds analyzed in the cotyledon and in the seed coat in the Spanish bean core collection of beans.

	Cotyledon components					Seed coat components				
	Protein	Starch	Amylose	Amylopectin	Apparent	Ashes	Ca	Dietary	Mg	Uronic
					amylose			fiber		acids
Mean	232.48	396.05	263.01	407.26	295.71	53.08	13.02	710.06	2.64	122.53
SD	25.49	21.19	22.60	35.42	18.30	11.32	5.09	72.36	0.42	10.18
SEM	1.83	1.52	1.62	2.54	1.31	0.81	0.36	5.18	0.03	0.73
Minimum	192.38	339.64	208.00	333.67	241.33	34.26	5.15	554.49	1.97	95.43
Maximum	304.24	446.53	291.00	482.00	332.81	94.27	30.74	911.22	4.47	154.77
CV	10.97	5.35	8.59	8.70	6.19	21.33	39.07	10.19	15.99	8.31

*Each accession was analyzed per triplicate. Values are expressed in g/kg dry matter. SD, standard deviation; SEM, standard error of the mean; CV, coefficient of variation (in %).*

Mean phenotypic values were used to estimate genotypic correlations among chemical compounds (Table 2). In the cotyledon components, negative correlations were identified between protein and starch (-0.60, *p* < 0.001), amylose (-0.37, *p* < 0.001), and apparent amylose (-0.34, *p* < 0.01). Positive correlations were identified between many other traits, the most significant being between amylose and apparent amylose (0.69, *p* < 0.001), and between starch and amylose (0.5, *p* < 0.001). With regard to the seed coat composition, several significant correlations were found, including between Ca and ashes (0.91, *p* < 0.001), uronic acid and dietary fiber (0.42, *p* < 0.001), and uronic acid and ashes (0.35, *p* < 0.01). In relation to correlations between seed coat and cotyledon compounds, no correlations were identified. Considering the wide range of genetic diversity explored, we also performed a correlation analysis between the chemical results obtained in this work and both sensory and culinary traits reported previously in the same samples (Rivera et al., 2016). This analysis included 174 out of the original 195 accessions, because for some accessions there were not enough available seeds to perform the sensory and culinary analysis, for which a large sample is needed. Several significant correlations were identified (Table 2). With regard to sensory traits, the most significant correlations were found between mealiness and the seed coat measurements of ashes, Ca, dietary fiber, and uronic acid (-0.62, -0.57, -0.42, and -0.47, *p* < 0.001, respectively), and between seed coat brightness and ashes, Ca, dietary fiber, and uronic acid (0.52, 047, 0.43, and 0.46, *p* < 0.001, respectively). Fewer significant correlations were found with the culinary traits, for instance between rate of water absorption and ashes, Ca, dietary fiber, and uronic acid (0.39, 0.36, *p* < 0.001; 0.33 *p* < 0.01; and 0.3, *p* < 0.05, respectively), and between cooking time and protein (0.33, *p* < 0.01). Percentage of white surface color was positively correlated with all chemical components measured in the seed coat, while 100 seed weight correlated only with Ca (-0.37, *p* < 0.001) and ashes (-0.34, *p* < 0.01) (Table 2).

**Table 2:** Genotypic correlations (Pearson coefficient with Bonferroni correction) between chemical composition traits measured in the cotyledon and seed coat, and sensory and culinary traits measured in a previous study (Rivera et al., 2016) using the same samples.

	Protein	Starch	Amylose	Amylopectin	Apparent	Ashes	Ca	Dietary	Mg	Uronic
					amylose			fiber		acids
Seed coat brightness	–0.08	0.04	0.11	0.12	0.15	0.52***	0.47***	0.43***	0.22	0.46***
Seed coat roughness	0.14	–0.3*	–0.11	–0.03	–0.05	0.37***	0.38***	0.22	0.2	0.25
Seed coat perception	–0.15	0.27	0.21	–0.02	0.32**	–0.29	–0.28	–0.19	0.01	–0.11
Mealiness	–0.06	0.2	0.09	–0.11	0.09	–0.62***	–0.57***	–0.42***	–0.15	–0.47***
Flavor	–0.03	0.26	0.21	–0.12	0.22	–0.35**	–0.3*	–0.18	–0.03	–0.16
Aroma	–0.07	0.37***	0.28	–0.12	0.23	–0.35**	–0.33**	–0.26	–0.01	–0.13
Rate of water absorption	0.04	–0.11	–0.07	0.06	–0.09	0.39***	0.36***	0.33**	0	0.3*
Water absorption during cooking (%)	0.21	–0.11	–0.02	–0.12	–0.03	0.25	0.21	0.16	0.11	0.17
Cooking time (min)	0.33**	–0.14	–0.09	–0.13	–0.04	0.06	0.08	0.02	0.1	0
Whole beans (%)	0.09	–0.11	–0.04	0.01	–0.24	–0.21	–0.2	0.06	–0.06	–0.11
White surface color (%)	0.05	–0.03	–0.02	0.07	0.03	0.57***	0.52***	0.47***	0.33***	0.53***
100 seed weight (g)	–0.06	0.13	0.09	–0.09	0.07	–0.34**	–0.37***	0.03	0	0.01
Protein		–0.6***	–0.37***	–0.22	–0.34**	0.05	0.08	–0.09	0.1	–0.15
Starch			0.5***	0.24	0.44***	–0.13	–0.1	0.03	–0.16	–0.04
Amylose				0.33*	0.69***	–0.08	–0.02	–0.06	–0.03	–0.13
Amylopectin					0.33**	0.09	0.07	–0.03	–0.02	–0.05
Apparent amylose							–0.01	0.06	–0.21	0.02
Ashes							0.91***	0.27	0.09	0.35**
Ca								0.2	0	0.21
Dietary fiber									0.06	0.42***
Mg										0.25

*Each accession was analyzed per triplicate. ^∗∗∗^*p* < 0.001; ^∗∗^*p* < 0.01; ^∗^*p* < 0.05.*

### Effect of Seed Color on Chemical Variability

**FIGURE 2:**
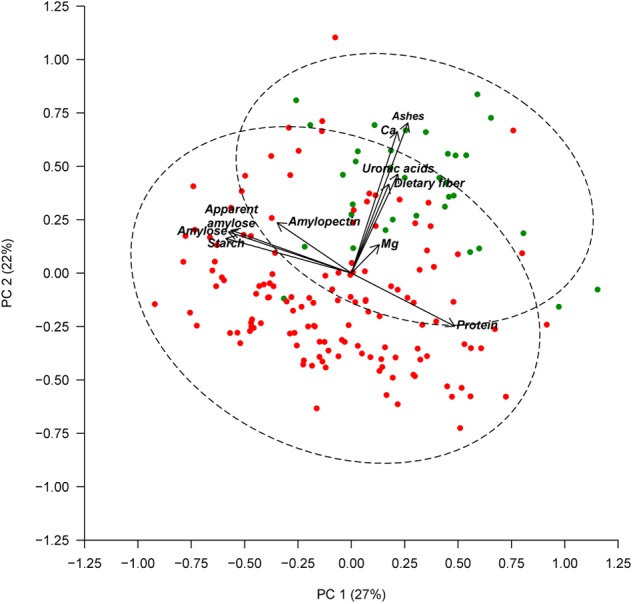
Principal component analysis based on chemical composition of the seed coat and cotyledon. Accessions with white seeds are represented with green symbols and accessions with colored seeds are represented with red symbols. The dashed lines represent the 95% confidence ellipses.

Seed color description, as reported in Rivera et al. (2016), revealed that 38 out of the 174 accessions studied had white seeds, and 136 accessions presented colored seeds. An ANOVA comparing the seed color classification (white or colored) was performed for all of the chemical traits measured (Table 3). Significant differences were found between white and colored seeds for all of the traits measured in the seed coat (ash, Ca, dietary fiber, Mg, and uronic acid), while solely for starch content regarding the traits measured in the cotyledon. White seeds tended to have higher concentrations for all of the traits measured in the seed coat and had lower starch content. The PCA created, considering all of the chemical traits evaluated in the seed coat and cotyledon with the different representation of the white and colored accessions, showed that the distribution of the accessions along the first (PC1) and second (PC2) principal components together explain 49% of the total variation (Figure 2). PCA showed that ash, Ca, uronic acids, and dietary fiber, which were correlated with PC2, had the higher contribution for clustering between groups, while the other chemical compounds had a low influence. Nevertheless, the confidence intervals for the two color groups overlapped, highlighting that within the colored group, there are genotypes with high values for the chemical traits analyzed.

**Table 3:** Analysis of variance to compare the levels of the different chemical components measured in the cotyledon and seed coat according to seed coat color classification proposed in Rivera et al. (2016).

	Cotyledon components					Seed coat components				
	Protein	Starch	Amylose	Amylopectin	Apparent	Ashes	Ca	Dietary	Mg	Uronic
					amylose			fiber		acids
	
Significance	ns	^∗∗^	ns	ns	ns	^∗∗∗^	^∗∗∗^	^∗∗∗^	^∗∗^	^∗∗∗^
White seeds	236.10	387.87	256.50	405.90	293.10	65.71	17.84	774.51	2.82	131.30
Colored seeds	229.80	399.46	264.60	406.48	296.63	50.61	11.91	696.47	2.59	120.34

*^∗∗∗^*p* < 0.001; ^∗∗^*p* < 0.01; ns, not significant. White seeds, 38 accessions; colored seeds, 136 accessions. Each accession was analyzed per triplicate. Values are expressed in g/kg.*

### Effect of Andean or Middle American Gene Pool on Chemical Variability

**FIGURE 3:**
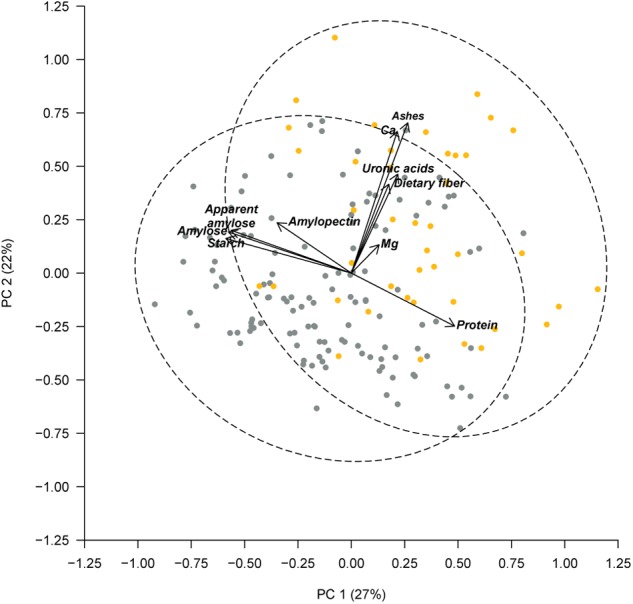
Principal component analysis based on chemical composition of the seed coat and cotyledon, considering the gene pool classification reported by PérezVega et al. (2009). Accessions with Middle American origin are represented by yellow symbols and accessions with Andean classification are represented by gray symbols. The dashed lines represent the 95% confidence ellipses.

Previously, PérezVega et al. (2009) had used phaseolin and molecular markers to classify the Spanish core collection according to their origin gene pool. Forty three out of the 174 accessions studied were classified within the Middle American gene pool, and 131 within the Andean gene pool. Using this classification, we performed an ANOVA, which revealed significant differences among gene pools for the following components: in the cotyledon, protein, starch (*p* < 0.001), amylose, and apparent amylose (*p* < 0.01); in the seed coat, ashes, and Ca (*p* < 0.001) (Table 4). Accessions classified in the Middle American gene pool tended to have higher ash, Ca, and protein contents, with lower levels of starch, amylose, and apparent amylose. The greatest difference was found for the content of Ca in the seed coat, with Middle American samples yielding a content 65% higher than Andean materials (Table 4). The same PCA as Figure 2 performed considering all of the chemical traits measured in the seed coat and cotyledon, but with the different representation of the accessions of Middle American and Andean gene pool showed that ash and Ca, which were correlated with PC2, had the higher contribution for clustering between groups (Figure 3). This multivariate analysis highlights that within the Andean gene pool there are genotypes with high values for uronic acids and dietary fiber contents (11 and 14 out of the 20 higher scores for uronic acids and dietary fiber, respectively, belong to accessions from Andean gene pool).

**Table 4:** Analysis of variance comparing the content of the different analytes studied according to the gene pool classification reported by PérezVega et al. (2009).

	Cotyledon components					Seed coat components				
	Protein	Starch	Amylose	Amylopectin	Apparent	Ashes	Ca	Dietary	Mg	Uronic
					amylose			fiber		acids
	
Significance	^∗∗∗^	^∗∗∗^	^∗∗^	ns	^∗∗^	^∗∗∗^	^∗∗∗^	ns	ns	ns
Middle American	242.22	384.35	253.89	407.64	289.52	65.67	18.57	710.98	2.72	123.76
Andean	227.11	401.45	266.04	406.69	298.21	49.70	11.31	711.88	2.60	122.24

*^∗∗∗^*p* < 0.001; ^∗∗^*p* < 0.01; ns, not significant. Forty-three accessions classified within the Middle American gene pool, and 131 within the Andean gene pool. Each accession was analyzed per triplicate. Values are expressed in g/kg dry matter.*

## Discussion

Use of NIR in this study enabled significant, in depth evaluation of the chemical composition of the Spanish core collection of beans. Data obtained here have added further knowledge to previously reported findings, helping make this one of the most well studied and characterized bean and legume collections. Detailed information, including pictures of seeds and flowers for each accession can be found on the website of the Spanish National Plant Genetic Resources Center: http://www.crf.inia.es/crfesp/paginaprincipaljudia.asp (verified 20 June 2018).

The chemical analysis performed on 20 samples enabled the inclusion of new reference values to the NIR models (Plans et al., 2012, 2013), thus increasing their robustness, as described by Barton et al. (2000), who showed the effect of adding new samples (∼10) to the memory based models reduced ¼ the SEP of the predictions (Barton et al., 2000). Accordingly, the prediction of all the components with the improved NIR models showed good precision, as for all the traits under study GH was below 3, which has been described as the threshold for considering a prediction robust (Shenk et al., 1997).

All the accessions showed a good agronomic behavior, completing the crop cycle, and enabling the harvesting of seeds. This was probably because all the accessions are landraces originating in similar agroclimatic conditions to those of the experimental field, thus yielding a similar phenotype to that of their area of origin. We identified great variability among the accessions of the core collection in some important chemical traits analyzed in the seed coats and cotyledons. The coefficients of variation were low in some cases [e.g., starch, amylose, amylopectin, apparent amylose, and uronic acid all had CV below 10% (Table 1)]. However, even in these cases, the extreme values were found to be far apart (with the maximum value at least 30% higher than the minimum for all of these traits). The proportion of seed coat ranged from 4% to 10%, CV (11.45%). These results are in accordance with those reported by Singh et al. (1968) and PérezVega et al. (2009), and slightly different from the variability described by Moraghan et al. (2006).

The few studies that have analyzed the chemical composition of the seed coat and cotyledon separately have reported values within the ranges found in this study (Singh et al., 1968; Moraghan et al., 2006). We were unable to find other studies that differentiated between components in the seed coat and cotyledon, but as the seed coat represents only a small proportion of the total weight of the beans, we can assume that the amounts found for the analytes in the cotyledon are not very different from what would be found in an analysis of the entire seed. The genetic differences identified for proteins and carbohydrates are within the range reported in studies considering the whole species (Sathe et al., 1984a,b; Hayat et al., 2014), and somewhat larger than those reported in specific collections (Paredes et al., 2009). Thus, it seems that the Spanish core collection represents a rich source of genetic variation for chemical composition in beans. Moreover, this diversity is present in genotypes that show good adaptation to the agroclimatic conditions of the Iberian Peninsula. Considering that this collection has been characterized by several traits, specific accessions can be selected for their agronomic, chemical, and sensory profiles, and used as elite genotypes to perform breeding programs devoted to obtain new varieties with good agronomic behavior, high contents of nutritional compounds, and sensory profiles close to consumer demands. This strategy can promote the consumption of this legume and achieve the objective of increasing the proportion of vegetable protein in the human diet.

With the aim of determining whether correlations exist between the assessed parameters which could potentially limit the progress in breeding programs for quality in common beans, we conducted a correlation analysis considering all of the traits studied in the cotyledon and seed coat. In this study, we identified significant positive correlations among the analyzed compounds in the seed coat, such as Ca and ash (0.92, *p* < 0.001). Moreover, results showed a significant correlation between protein and carbohydrates. These results are in accordance with Vargas-Torres et al. (2004) and Casañas et al. (2013), and imply that an increase in the concentration of protein leads to a decrease in starch content. No correlations have been found between the chemical composition of the seed coat and cotyledon, signaling that the chemical composition of both parts seems to be under independent genetic control. In accordance with this hypothesis, Casañas et al. (2013) identified independent quantitative trait loci (QTL) controlling chemical composition of the seed coat and cotyledon.

With regards to the relationship between chemical composition and sensory characteristics, it seems that ashes and Ca are the components with the strongest influence. Relative to the culinary traits, some authors have reported a tendency for genotypes with shorter cooking times to retain higher nutrient concentrations than those with longer cooking times (Wiesinger et al., 2016). In this work, the only relationship with cooking time was observed for protein. The lack of data for phytate content in the collection is an important shortcoming to complete this analysis, as this compound shows a strong relation with the culinary or sensory traits in beans (Casañas et al., 2002; Coelho et al., 2007). Our results also showed a negative correlation between Ca and weight of 100 seeds (-0.37, *p* < 0.001). In a study of eight accessions, Moraghan and Grafton (2001) reported a much higher negative correlation between these two characteristics. However, the diversity in Ca concentration observed in some accessions with a similar 100 seed weight highlights that there are other factors involved in determining this characteristic, such as the color of the seeds (Table 2) or the seed coat area/cotyledon weight ratios (Moraghan and Grafton, 2001).

Although there were many significant genotypic correlations between chemical components and culinary or sensory traits, most were not strong enough to make indirect selection efficient (i.e., the selection for a desired trait using another trait that is genetically linked). These findings contrast with those of studies examining smaller collections, especially those done within a single variety or a few varieties, which found genetic correlations between chemical components and sensory traits (Casañas et al., 2006; Rivera et al., 2015). Therefore, in a large collection with greater variability, these effects are understandably diluted. Moreover, considering the important GxE effects on quantitative traits, and more specifically on traits related to chemical composition (Shellie and Hosfield, 1991; Moraghan and Grafton, 2001; Florez et al., 2009) and sensory profile (Romero del Castillo et al., 2008), significant correlations should be validated in further studies considering different agroclimatic conditions. For instance, in our case, we have performed the experiment in a field with high content of Ca and low content of P. Soil composition influences the chemical composition of common beans (Sameni et al., 1980; Moraghan and Grafton, 2001). Thus new experiments should be conducted in sites with highly different edaphologic characteristics to complement this study.

Seed coat color in common beans is controlled by several loci that act independently or in an epistatic manner to affect the color and pattern (Moghaddam et al., 2014). The P locus is known as the core factor for all seed coat color genotypes. The presence of its recessive allele results in white seeds and flowers due to its epistatic effect on the expression of the other color and pattern genes (Bassett, 2007). The background color and different patterns of marking on the seed coat are caused by the accumulation of anthocyanin and phenolic substances, which influence nutritional value and are coded by various gene systems (Beninger et al., 1999, 2000; Caldas and Blair, 2009). Our results show that there are significant differences in seed coat composition (ash, Ca, dietary fiber, Mg, and uronic acid) between white and colored seeds, but no differences for cotyledon composition (except for starch) (Table 3 and Figure 2). Although the negative correlation between starch and protein (*r* = -0.60, *p* < 0.001), which was confirmed within both groups (white seeds, *r* = -0.53, *p* < 0.01; colored seeds, *r* = -0.60, *p* < 0.001), the significant differences between color groups for the starch are not reflected in the protein content. Mean values for protein content in colored accessions was lower with respect to white seeded, but these differences were not statically supported, mainly because the variation within groups was very high for this trait. In contrast, other authors found greater amounts of protein and other nutrients in the whole seeds of black beans compared to lighter varieties (Silva et al., 2012; Hacisalihoglu and Settles, 2013). Our results concur with the results reported by Casañas et al. (2013), who reported five QTL associated with ash, Ca, dietary fiber, Mg, and uronic acid content, which were mapped in the region of the P gene.

Common beans were independently domesticated in the Andean and Middle American areas. Many authors have studied this phenomenon and attributed differentiated characteristics to beans originating from each area (Gepts and Debouck, 1991; Singh et al., 1991; Schmutz et al., 2014), but the differences for chemical composition have been scarcely studied. Research performed by PérezVega et al. (2009), comparing molecular markers and phaseolins in the Spanish core collection of common bean, enabled the classification of accessions into the Middle American or Andean gene pool. Following this classification, we performed an ANOVA that showed significant differences for all chemical components in the cotyledon, with the exception of amylopectin, and significant differences only for ash and Ca in the seed coat (Table 4 and Figure 3). Considering that phaseolins are the major seed storage protein (constitute over 50% of total protein in beans), and that the concentration and type of phaseolin present contribute to the classification gene pool (Gepts et al., 1986; Singh et al., 1991), it is to be expected that cotyledon components are those with the greatest differences depending on gene pool. Results obtained from our study indicate that seeds from the Spanish core collection identified as of the Middle American gene pool have a higher concentration of protein, ash, and Ca, and lower concentrations of carbohydrates. Some of these results are in accordance with those obtained by Islam et al. (2002). In their study on the CIAT core collection of beans, they looked at these and other chemical components in whole seeds from the two major gene pools (Middle American and Andean), as well as from the North Andean Group and a mixed group. Their study showed that accessions from the Middle American gene pool contained higher Ca concentrations than those from the North Andean and Andean gene pools. However, their study also reported a lower concentration of phaseolin protein in the Middle American gene pool.

## Conclusion

In conclusion, results of this work show that the variability in the Iberian Peninsula, a second center of diversity for *P. vulgaris*, is very high, and can be used as an important source for breeding more nutritional and palatable varieties or directly by farmers. Moreover, our results point out that there are not strong correlations between the most important nutritional and sensory attributes, which is an important finding, signaling that synchronic improvement of both traits is feasible. Significant differences for nutritional composition have been identified between colored and non-colored seeds and between gene pools (Middle American and Mesoamerican), contributing to the knowledge about the diversification process of this species. NIRS models improved in this work can be an useful technology for mass phenotyping of other sources of genetic diversity within the species.

## Author Contributions

FC, AR, MP, and JSa planned the study. AR, AR, and JSa conducted the experiments. MP carried out the improvement of models and obtained the data. AR, FC, MP, and JC wrote the manuscript. JSi revised the article critically. All authors read and approved the final manuscript.

## Conflict of Interest Statement

The authors declare that the research was conducted in the absence of any commercial or financial relationships that could be construed as a potential conflict of interest.
